# The different subtelomeric structure among 1RS arms in wheat-rye 1BL.1RS translocations affecting their meiotic recombination and inducing their structural variation

**DOI:** 10.1186/s12864-023-09525-9

**Published:** 2023-08-11

**Authors:** Ziying Xiong, Jie Luo, Yang Zou, Qilin Tang, Shulan Fu, Zongxiang Tang

**Affiliations:** 1 State Key Laboratory of Crop Gene Exploration and Utilization in Southwest China, Sichuan Agricultural University, Chengdu, 611130 China; 2 College of Agronomy, Sichuan Agricultural University, Chengdu, 611130 China; 3 Maize Research Institute, Sichuan Agricultural University, Chengdu, 611130 China

**Keywords:** Wheat, Rye, 1BL.1RS, Tandem repeat, Unequal recombination

## Abstract

**Background:**

The 1RS arm of wheat-rye 1BL.1RS translocations contains several subtelomeric tandem repeat families. To study the effect of the difference in the composition of these tandem repeats on the meiotic recombination of 1RS arms can help to enrich the genetic diversity of 1BL.1RS translocation chromosomes.

**Results:**

Five wheat-rye 1BL.1RS translocation cultivars/lines were used to build two cross combinations including group 1 (20T401 × Zhou 8425B, 20T401 × Lovrin 10 and 20T401 × Chuannong 17) and group 2 (20T360-2 × Zhou 8425B, 20T360-2 × Lovrin 10 and 20T360-2 × Chuannong 17). Oligonucleotide (oligo) probes Oligo-s120.3, Oligo-TR72, and Oligo-119.2-2 produced the same signal pattern on the 1RS arms in lines 20T401 and 20T360-2, and another signal pattern in the three cultivars Zhou 8425B, Lovrin 10 and Chuannong 17. The Oligo-pSc200 signal disappeared from the 1RS arms of the line 20T401, and the signal intensity of this probe on the 1RS arms of the line 20T360-2 was weaker than that of the three cultivars. The five cultivars/lines had the same signal pattern of the probe Oligo-pSc250. The recombination rate of 1RS arms in group 1 was significantly lower than that in group 2. In the progenies from group 1, unequal meiotic recombination in the subtelomeric pSc119.2 and pSc250 tandem repeat regions, and a 1BL.1RS with inversion of 1RS segment between the pSc200 and the nucleolar organizer region were found.

**Conclusions:**

This study provides a visual tool to detect the meiotic recombination of 1RS arms. The meiotic recombination rate of 1RS arms was affected by the variation of pSc200 tandem repeat, indicating the similar composition of subtelomeric tandem repeats on these arms could increase their recombination rate. These results indicate that the 1RS subtelomeric structure will affect its recombination, and thus the localization of genes on 1RS by means of meiotic recombination might also be affected.

**Supplementary Information:**

The online version contains supplementary material available at 10.1186/s12864-023-09525-9.

## Background

Higher frequency of chromosome meiotic recombination can enrich the genetic diversity of crop breeding materials. A lot of genes that control chromosome meiotic recombination have been discovered [1]. The function of genes is based on the structure and organization of chromatin, and the frequency of chromosome meiotic recombination is also controlled by chromatin structure [2]. The complex cytological structure caused by tandem repeats could affect the meiotic recombination between wheat 5 A chromosomes [3, 4]. The correct recognition and pairing of homologous chromosomes is the prerequisite for their successful meiotic recombination and the effect of the subtelomeric regions of *Hordeum chilense*c chromosomes on their meiotic recognition and pairing was observed [5]. The wheat chromosomes with rye centromeres were used to indicate that the subtelomeric regions are responsible for the homologous recognition during meiosis [6]. Homoeologous *Hordeum* chromosomes can pair during early meiosis even in the wheat background with *Ph1* locus, and it was presumed that this was related to the DNA sequence(s) within the subtelomeric regions [7]. The molecular basis for the meiotic recognition and paring of homologous chromosomes in wheat might be the compositions of DNA sequences in the subtelomeric regions [8]. These previous studies indicate that the subtelomeric regions of chromosomes play an important role in chromosome recognition and pairing, and the same has already been reviewed [2].

According to these previous reports, it is worth studying whether the different composition of tandem repeats in subtelomeric regions affect meiotic recombination. Wheat-rye 1BL.1RS translocations are the suitable materials for this study because the subtelomeric region of 1RS arm contains several nonhomologous tandem repeats and they exhibit the complexity of organization [9, 10]. Although the meiotic paring of 1BL.1RS translocations was affected by the distinct subtelomeric structure of 1RS arms, it is unclear what kind of differences of tandem repeats can affect their meiotic recombination [11]. In this study, some F_1_ plants were obtained through the crosses using different 1BL.1RS translocation cultivars/lines as parents. Oligonucleotide (oligo) probes derived from pSc119.2 [12], pSc200, pSc250 [13], rDNA of 1R chromosome of rye Lo7 [14] and pTa-s120 [15] were used to investigate the structural variations of these F_1_ plants and the F_2_ plants. In addition, a new tandem repeat found in this study was also used to design an oligo probe and was used to investigate the structure of 1RS arms.

## Results

### Different structure of the 1BL.1RS chromosomes

**Fig. 1:**
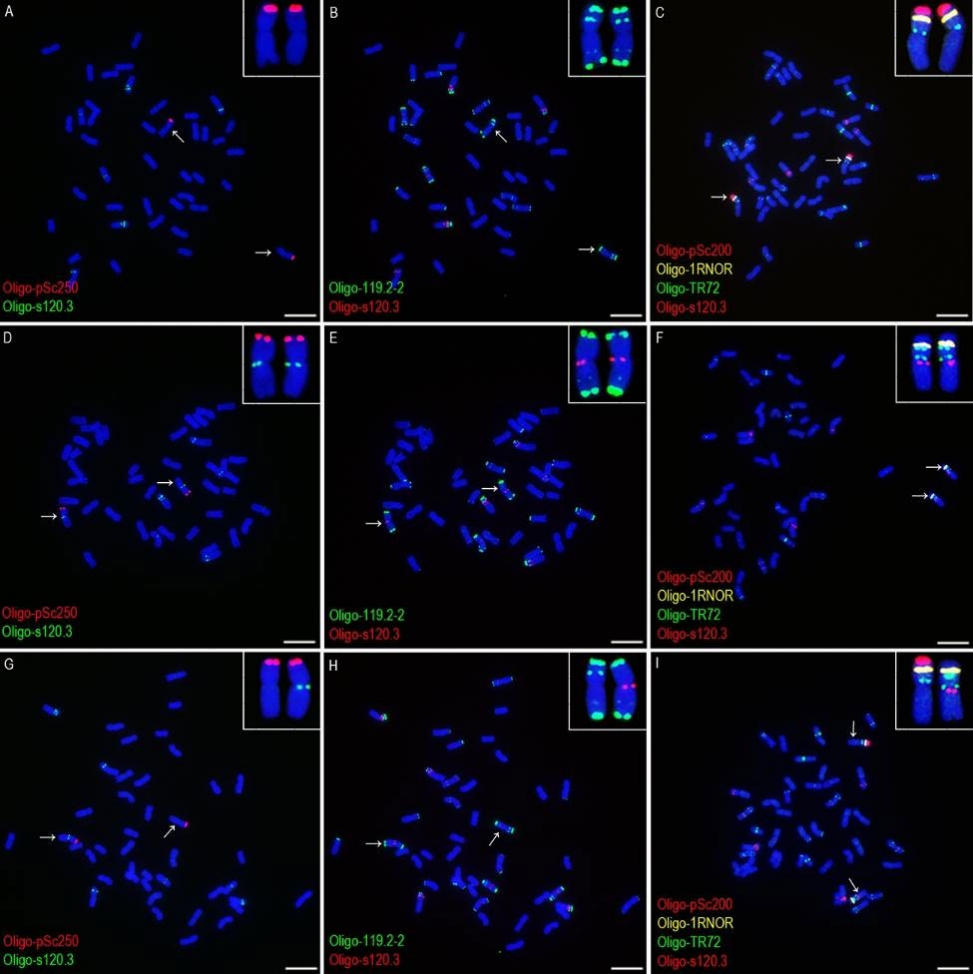
ND-FISH analysis of root-tip metaphase chromosomes of line 20T401, cultivar Lovirin 10 and the F_1_ plant representing the cross combination group 1. (**A-C**) The cells of Lovirin 10. (**A**) and (**B**) are the same cells. (**D-F**) The cells of 20T401. (**D**) and (**E**) are the same cells. (**G-I**) The cells of a F_1_ plant from 20T401 × Lovirin 10. (**G**) and (**H**) are the same cells. The probes Oligo-pSc200 (red), Oligo-pSc250 (red), Oligo-119.2-2 (green), Oligo-1RNOR (yellow), Oligo-TR72 (green) and Oligo-s120.3 (red or green) are marked in the figure. The arrows indicate the 1BL.1RS translocation chromosomes and the inserts indicate the enlarged ones. Chromosomes were counterstained with DAPI (blue). Scale bar: 10 μm

**Fig. 2:**
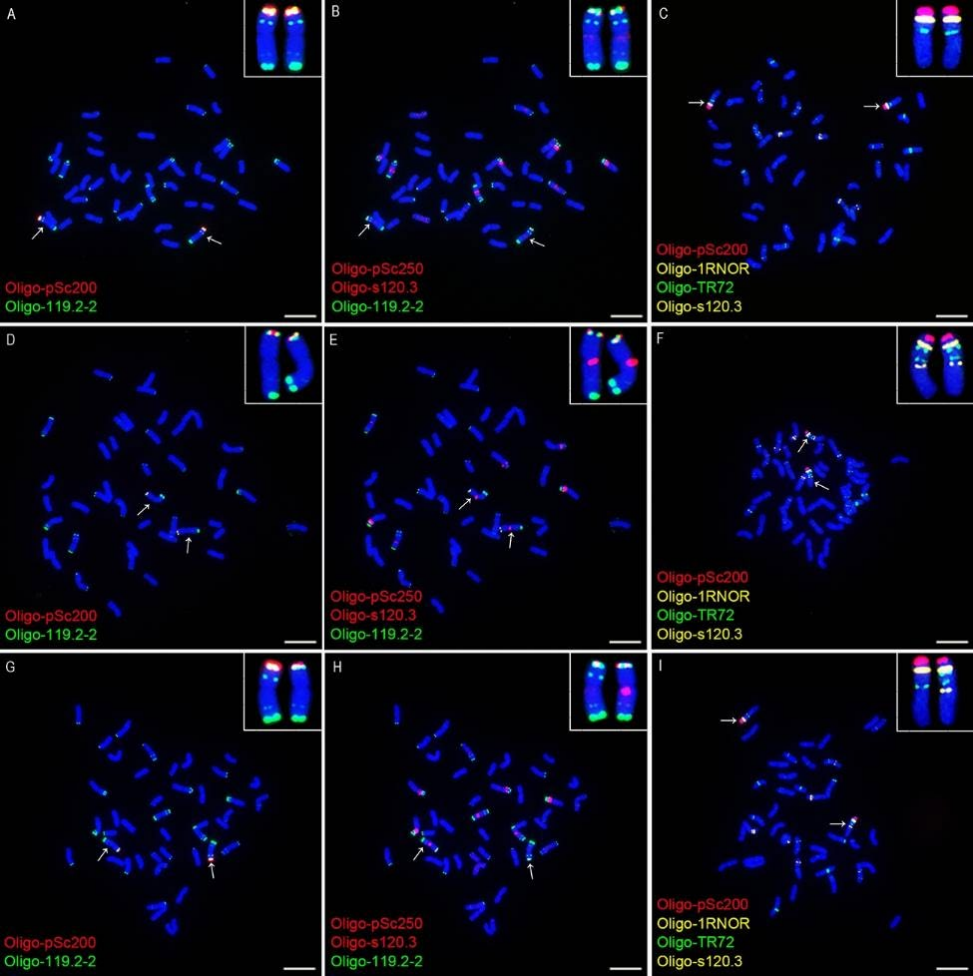
ND-FISH analysis of root-tip metaphase chromosomes of line 20T360-2, cultivar Zhou 8425B and the F_1_ plant representing the cross combination group 2. (**A-C**) The cells of Zhou 8425B. (**A**) and (**B**) are the same cells. (**D-F**) The cells of 20T360-2. (**D**) and (**E**) are the same cells. (**G-I**) The cells of a F_1_ plant from 20T360-2 × Zhou 8425B. (**G**) and (**H**) are the same cells. The probes Oligo-pSc200 (red), Oligo-pSc250 (red), Oligo-119.2-2 (green), Oligo-1RNOR (yellow), Oligo-TR72 (green) and Oligo-s120.3 (red or yellow) are marked in the figure. The arrows indicate the 1BL.1RS translocation chromosomes and the inserts indicate the enlarged ones. Chromosomes were counterstained with DAPI (blue). Scale bar: 10 μm

The wheat-rye 1BL.1RS translocation chromosomes can be identified using probes Oligo-119.2-2, Oligo-pSc200, Oligo-pSc250, and Oligo-1RNOR (Figs. 1 and 2). The pericentromeric regions of the 1BL arms in lines 20T360-2 and 20T401 contained the signal of Oligo-s120.3 and the 1BL arms in cultivars Zhou 8425B, Lovrin 10 and Chuannong 17 did not have this signal (Figs. 1 and 2). The probe Oligo-119.2-2 produced signals in the telomeric and intercalary regions of the 1RS arms in the cultivars Zhou 8425B, Lovrin 10 and Chuannong 17, and the telomeric signal is significantly stronger than the intercalary one (Figs. 1 and 2). The intercalary Oligo-119.2-2 signal disappeared from the 1RS arms in the lines 20T360-2 and 20T401 (Figs. 1 and 2). The probe Oligo-TR72 produced signals in the pericentromeric and intercalary regions of the 1RS arms in the lines 20T360-2 and 20T401, and the 1RS arms in Zhou 8425B, Lovrin 10 and Chuannong 17 only contained its pericentromeric signal (Figs. 1 and 2). The signal of Oligo-pSc200 appeared on the 1RS arms in the line 20T360-2 and the cultivars Zhou 8425B, Lovrin 10 and Chuannong 17, but it disappeared from the line 20T401 (Figs. 1 and 2). All the 1RS arms in the five 1BL.1RS translocations contained the signals of Oligo-pSc250 and Oligo-1RNOR, and they did not display variation (Figs. 1 and 2).

The root-tip metaphase chromosomes of the F_1_ plants from the six cross combinations in the group 1 and group 2 were used for ND-FISH analysis using the six oligo probes. In the three kinds of F_1_ plants in group 1, the 1BL.1RS chromosome without Oligo-pSc200 was derived from the line 20T401 (Fig. 1I). On this translocation chromosome, the 1BL arm carried Oligo-s120.3 signal, and the 1RS arm carried the pericentromeric and intercalary signals of Oligo-TR72, the telomeric Oligo-119.2-2 signal and the Oligo-pSc250 signal (Fig. 1G-I). The intensity of the Oligo-pSc250 signal and the telomeric Oligo-119.2-2 signal had no difference between the 1RS arms in the line 20T401 and those in the cultivars Zhou 8425B, Lovirin 10 and Chuannong 17 (Fig. 1G, H). In the three kinds of F_1_ plants in group 2, the 1BL.1RS chromosome with the Oligo-s120.3 signal, and the pericentromeric and intercalary signals of Oligo-TR72 was from the line 20T360-2 (Fig. 2H, I). The obvious difference in the signal intensity of Oligo-pSc200 was observed between the 1RS arm in the line 20T360-2 and those in the cultivars Zhou 8425B, Lovirin 10 and Chuannong 17 (Fig. 2G, I), and no difference was observed for the signal strength of Oligo-pSc250 and the telomeric Oligo-119.2-2 signal (Fig. 2G, H). The signal patterns of four oligo probes that displayed diversities of 1BL.1RS chromosomes are included in Table 1. The signal patterns of Oligo-pSc250 and Oligo-1RNOR are not listed because they were not variable among the five 1BL.1RS translocations.

**Table 1:** The signal patterns of four oligo probes on 1BL.1RS chromosomes in the F_1_ plants

Probes	F_1_ plants in group 1		F_1_ plants in group 2	
	1BL.1RS in line 20T401	1BL.1RS in three cultivars	1BL.1RS in line 20T360-2	1BL.1RS in three cultivars
Oligo-s120.3	1BL^Per^	No signal	1BL^Per^	No signal
Oligo-119.2-2	1RS^Tel^	1RS^Tel^; 1RS^Int^	1RS^Tel^	1RS^Tel^; 1RS^Int^
Oligo-TR72	1RS^Per^; 1RS^Int^	1RS^Per^	1RS^Per^; 1RS^Int^	1RS^Per^
Oligo-pSc200	No signal	1RS^Tel^	1RS^Tel^ (weak)	1RS^Tel^ (strong)

Three cultivars are Zhou 8425B, Lovirin 10 and Chuannong 17. “1BL^Per^” indicates the signal on the pericentromeric region of 1BL arm. “1RS^Tel^”, “1RS^Int^” and “1RS^Per^” indicate the signal on the telomeric, intercalary and pericentromeric regions of 1RS arm, respectively. “weak and strong” indicates that the signal intensity of Oligo-pSc200 on the 1RS arm from line 20T360-2 was weaker than that from the three cultivars, and vice versa

### Meiotic recombination of 1RS arms

**Fig. 3:**
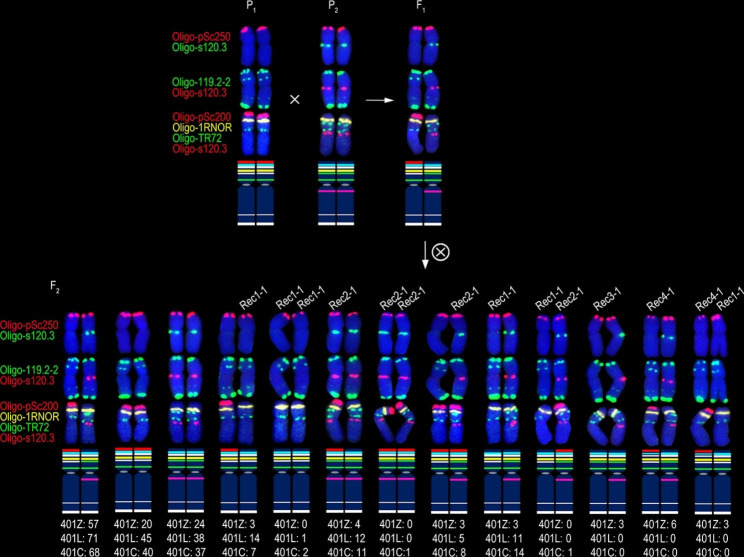
Types of 1BL.1RS translocation chromosomes in the F_2_ plants from the cross combination group 1. “P_1_” and “P_2_” indicate the chromosomes in parental plants. “F_1_” indicates the chromosomes in F_1_ generation plant. “×” in the circle indicates selfing. “F_2_” indicates the chromosomes in F_2_ generation plants. “Rec1-1”, “Rec2-1”, “Rec3-1” and “Rec4-1” indicate the recombinant 1BL.1RS translocations from cross combination group 1. “401Z: 57”, “401L: 71” and “401C: 68” indicate there are 57, 71 and 68 F_2_ plants from 20T401 × Zhou 8425B, 20T401 × Lovirin 10 and 20T401 × Chuannong 17, respectively, containing the two types of 1BL.1RS, and so on. On the idiograms, the red, blue, white, yellow, green and pink bands represent the signals of Oligo-pSc200 (red), Oligo-pSc250 (red), Oligo-119.2-2 (green), Oligo-1RNOR (yellow), Oligo-TR72 (green) and Oligo-s120.3 (red or green), respectively

**Fig. 4:**
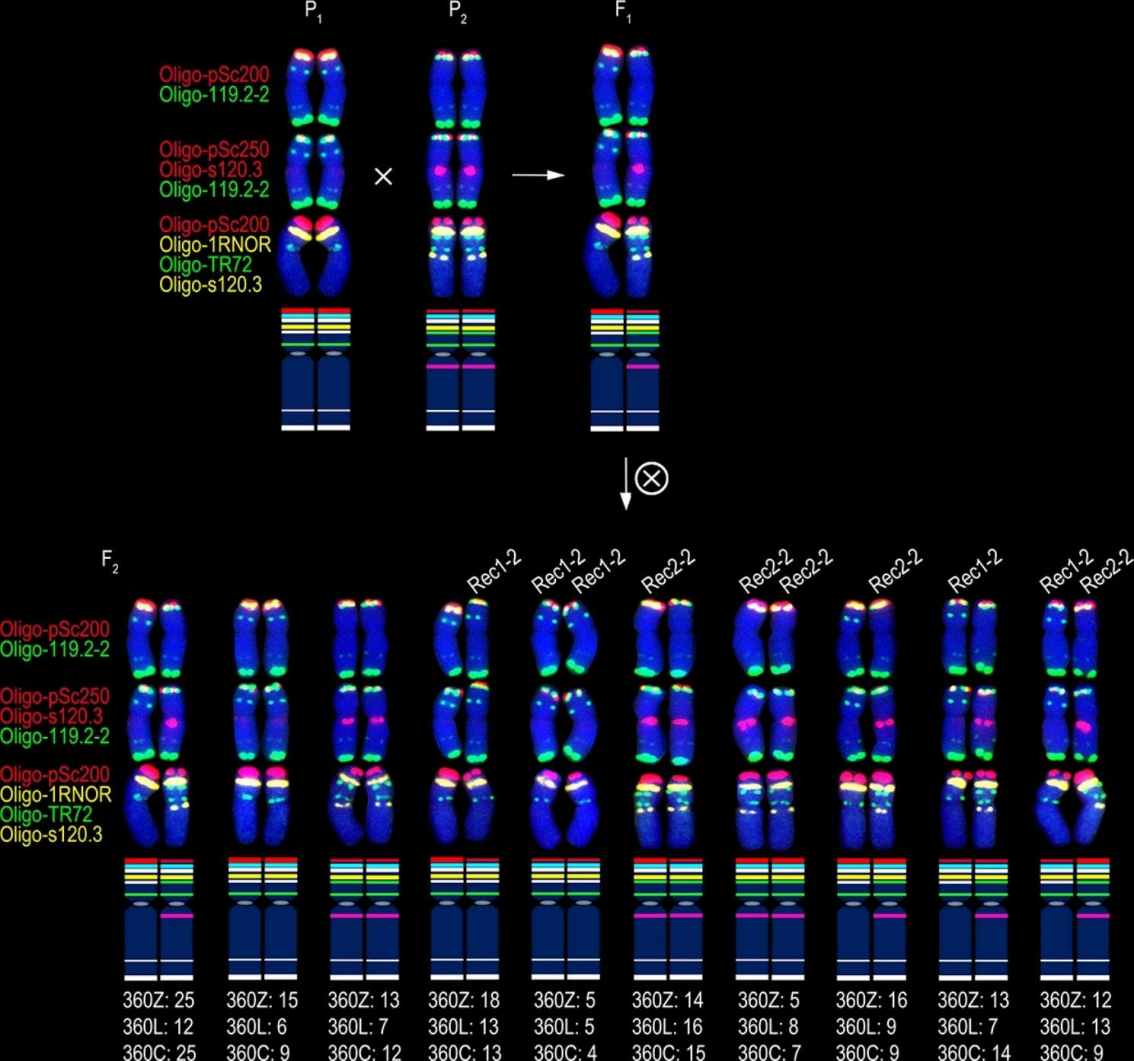
Types of 1BL.1RS translocation chromosomes in the F_2_ plants from the cross combination group 2. The mean of “P_1_”, “P_2_”, “F_1_”, “×” in the circle and “F_2_” is the same as that in Fig. 3. “Rec1-2” and “Rec2-2"indicate the recombinant 1BL.1RS translocations from cross combination group 2. “360Z: 25”, “360L: 12” and “360C: 25” indicate there are 25, 12 and 25 F_2_ plants from 20T360-2 × Zhou 8425B, 20T360-2 × Lovirin 10 and 20T360-2 × Chuannong 17, respectively, containing the two types of 1BL.1RS, and so on. On the idiograms, the red, blue, white, yellow, green and pink bands represent the signals of Oligo-pSc200 (red), Oligo-pSc250 (red), Oligo-119.2-2 (green), Oligo-1RNOR (yellow), Oligo-TR72 (green) and Oligo-s120.3 (red or yellow), respectively

**Fig. 5:**
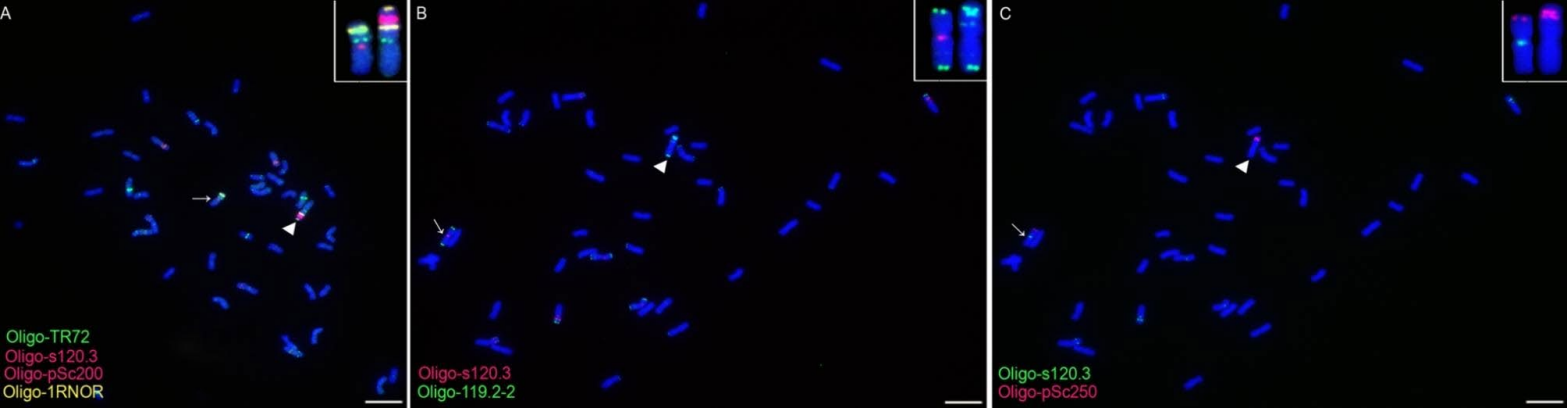
ND-FISH analysis of the root-tip metaphase chromosomes of the plant with an inversion 1RS. (**A-C**) Cells containing the 1BL.1RS translocation with an inversion 1RS segment, indicated by the triangles. (**B**) and (**C**) are the same cells. The probes Oligo-pSc200 (red), Oligo-pSc250 (red), Oligo-119.2-2 (green), Oligo-1RNOR (yellow), Oligo-TR72 (green) and Oligo-s120.3 (red or green) are marked in the figure. Arrows indicate the parental 1BL.1RS chromosomes and the inserts indicate the enlarged ones. Chromosomes were counterstained with DAPI (blue). Scale bar: 10 μm

One hundred and twenty-six, 197, 189, 136, 96 and 117 F_2_ generation seeds from the six cross combinations 401Z, 401L, 401C, 360Z, 360L and 360C were randomly selected, respectively, and they were analyzed to determine the recombination frequency of 1RS arms. The 512 F_2_ plants from 401Z, 401L and 401C (group 1) were divided into 13 types according to the structure of the 1BL.1RS translocation chromosomes in these plants (Fig. 3 and Additional files 1–4). Most of the 1024 1BL.1RS chromosomes were parental and F_1_ types (Fig. 3 and Additional file 1), and four kinds of recombinant 1RS arms were observed (Fig. 3 and Additional files 2–4). Recombinants Rec1-1 and Rec2-1 were derived from the recombination occurred in the interval between the signal sites of intercalary Oligo-TR72 (or intercalary Oligo-119.2-2) and Oligo-pSc200 (Fig. 3 and Additional files 2–4). The recombinant Rec3-1 was produced by the recombination in the interval between the intercalary and pericentromeric Oligo-TR72 signal sites, or between the signal sites of intercalary Oligo-119.2-2 and pericentromeric Oligo-TR72 (Fig. 3 and Additional file 4). In nine of the 126 F_2_ plants from the cross combination 401Z, the intensity of the Oligo-pSc250 and the telomeric Oligo-119.2-2 signals on the 1RS arm of the Rec4-1 was significant weaker than that of the other 1BL.1RS translocation (Fig. 3 and Additional file 4D-I). This result indicates that unequal recombination occurred in both the regions containing pSc250 and pSc119.2 tandem repeats because the intensity of these signals on the 1RS arms in the F_1_ plants was similar (Fig. 1G, H). The recombinant Rec4-1 only occurred in the progeny from the cross combination 401Z (Figs. 3 and 4). The recombination rates of 1RS arms from 401Z, 401L and 401C were 11.11% (28/252), 11.17% (44/394), and 12.70% (48/378), respectively (Fig. 3). One of the 197 F_2_ plants from the cross combination 401L contained a 1BL.1RS translocation with inversion of 1RS segment between the signal sites of Oligo-pSc200 and Oligo-1RNOR, leading to this 1RS arm containing a telomeric Oligo-1RNOR signal site, two telomeric Oligo-119.2-2 and two Oligo-pSc250 signal sites (Fig. 5). This 1RS arm with inversion was not included in the recombination frequency.

**Fig. 6:**
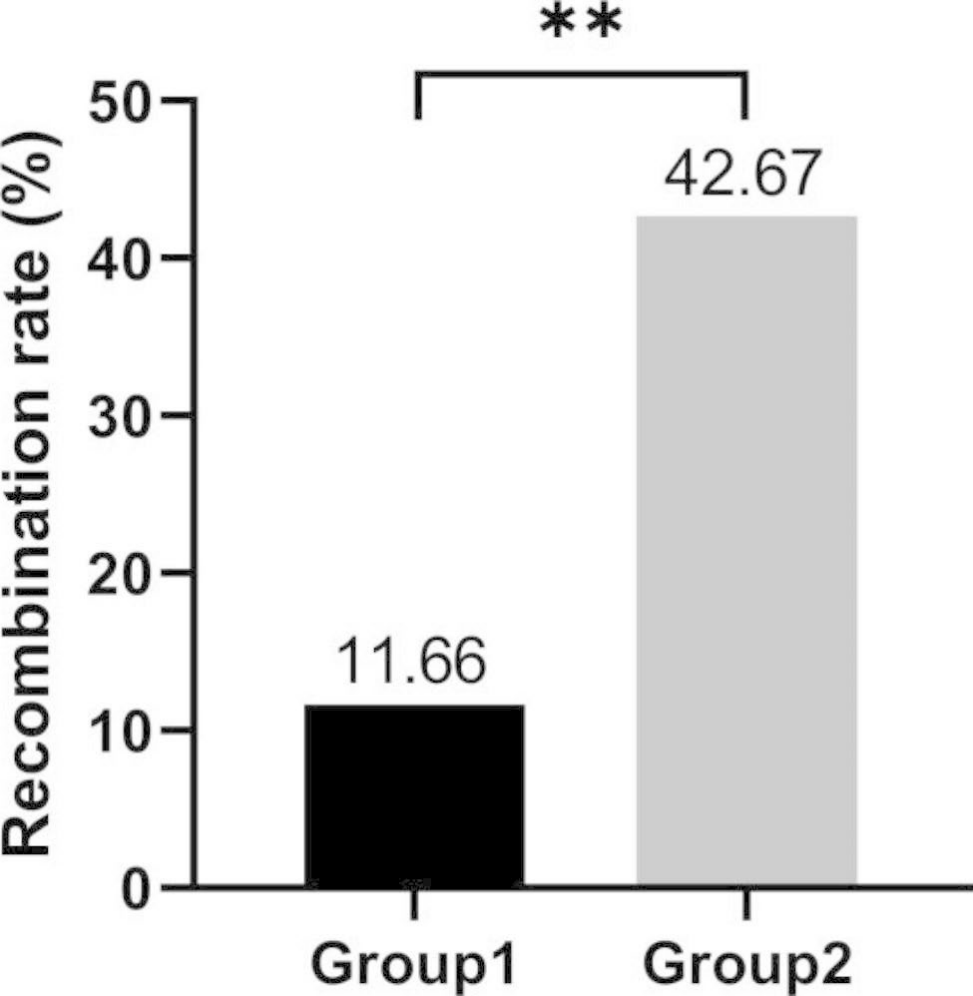
Comparing the recombination rate of 1RS arms between the cross combinations group1 and group 2. **: p < 0.01

According to the structure of 1BL.1RS translocation chromosomes, the 349 F_2_ plants from 360Z, 360L, and 360C (group 2) were divided into ten types (Fig. 4 and Additional files 5–7). Two kinds of recombinant 1RS arm were detected among the 698 1BL.1RS translocations and most of them were the parental and F_1_ types (Fig. 4 and Additional file 5). The recombinants Rec1-2 and Rec2-2 were produced by the recombination in the interval between the signal sites of intercalary Oligo-TR72 (or intercalary Oligo-119.2-2) and Oligo-pSc200 (Fig. 4 and Additional files 6, 7). The recombination rates of 1RS arms from 360Z, 360L and 360C were 38.60% (105/272), 50.52% (97/192), and 38.89% (91/234), respectively (Fig. 4). The unequal recombination of the tandem repeats was not detected among the F_2_ plants from the cross combination group 2. Significant difference of the average recombination rate on 1RS arm was observed between the progenies of cross combinations group1 and group 2 (Fig. 6).

For all the cross combinations, the recombination in the interval between the pericentromeric signal site of Oligo-TR72 and the signal site of Oligo-s120.3 was not considered because it is hard to happen in the region near the centromere. The double recombination cannot be determined, and the recombination rate was underestimated because of the lacking of enough cytological markers. In addition, in order to avoid the error caused by the inconsistent conditions of in situ hybridization and picture exposure times, only the heterozygotes were used to calculate the rate of unequal recombination in the tandem repeat regions, and the recombination rate was also underestimated. Therefore, more oligo probes that could reflect the structural polymorphism of 1BL.1RS translocations are needed and this will provide a convenient method to investigate the recombination between 1BL.1RS translocations.

## Discussion

### Enriching the polymorphism of 1RS in 1BL.1RS translocations

1RS-specific molecular markers and FISH technology using tandem repeats as probes were often used to investigate the polymorphism of 1RS arms in rye and wheat-rye 1BL.1RS translocations, and the rich polymorphism of 1RS arm in genus *Secale* was observed [10, 16–18]. However, narrow polymorphism of 1RS in 1BL.1RS and 1AL.1RS translocations was observed [10]. In this study, the two new oligo probes Oligo-119.2-2 and Oligo-TR72 displayed the new structural variations of 1RS arms. The 1RS arms in the line 20T401 do not contain the tandem repeat pSc200 and this is different from the reported 1BL.1RS and 1AL.1RS translocations [10]. The 1BL.1RS translocation lines 20T360-2 and 20T401 were derived from the octaploid triticale MK × common wheat hybrids. It has already been reported that triticale can be used to create translocations between rye and wheat chromosomes [19, 20]. The widespread whet-rye 1BL.1RS translocation in which the 1RS from the rye Petkus was selected from the progenies of triticale × wheat hybrids [21]. The frequency of the translocation between 1RS arm and wheat chromosomes was the highest among the progeny of octaploid tritlcale × common wheat hybrids [20]. Therefore, the 1RS in genus *Secale* should be fully used to create new 1BL.1RS translocations through triticale. However, the reason why the 1BL.1RS translocation was often found in the progenies of triticale × wheat hybrids is not clear. It might be due to the similar structure between the 1RS and 1BS arms, but evidence is needed to support this conjecture. In addition, the genetic diversity of 1BL.1RS translocations can also be enriched through the meiotic recombination between different 1RS arms [22–25]. Whereas the effect of the chromosomal structure on the meiotic recombination of homologous should be considered [26].

### The effecting of subtelomeric tandem repeats on the 1RS meiotic recombination

It has already been reported that the subtelomeric region of chromosome is important for the correct homologous chromosome recognition and pairing during meiosis [2, 5–8]. Rye 1RS arm is a good model for studying the effect of subtelomeric region on the meiotic paring and recombination because this region contains several tandem repeat families with abundant variations [9, 10]. In this study, the structural difference between the 1BL.1RS chromosomes in the lines 20T401 and 20T360-2 was displayed by the probe Oligo-pSc200. Compared with the three cultivars Zhou 8425B, Lovirin 10 and Chuannong 17, the two lines have the common difference displayed by the probes Oligo-s120.3, Oligo-TR72 and Oligo-119.2-2. So, for the cross combinations group 1 and group 2, only the structural difference displayed by the probe Oligo-pSc200 was considered. That is, according to the signal pattern of Oligo-pSc200, the structural difference between the 1RS arms in the cross combination group 1 was greater than that in the group 2, and this corresponded to the case that the recombination rate of 1RS arm in group 1 was lower than that in group 2. Although the other factors that affected the recombination of 1RS arms were not considered, the different composition of pSc200 tandem repeats should be one of the factors affecting the recombination of 1RS homologues because the variation trend of the recombination rate was similar among the three cross combinations in each group. Wheat 5 A chromosomes with different cytological structure also indicated that the smaller structural differences between chromosomes lead to a higher meiotic recombination frequency [3, 4].

Compared with the cross combination group 1, an increase in the copy number of the tandem repeat pSc200 in group 2 led to the significant increase in the recombination rate of 1RS arms in group 2. Four wheat-rye 1BL.1RS translocation lines in which the 1RS arms with distinct subtelomeric heterochromatin were used to investigate meiotic behavior of these arms, and the results indicated that the chromatin remodeling, which promotes homologous chromosome recognition and pairing, only occurred between the identical or nearly identical 1RS arms [11]. According to the results of the previous study, it can be presumed that an increase in the copy number of the tandem repeat pSc200 led to an increase in structural similarity between the subtelomeric regions of the two 1RS arms, thus increasing their recombination rate. This raises a question of whether the variation of the tandem repeats pSc119.2 and pSc250 can also affect the recombination. More 1RS variants are needed to answer this question. The recombination suppression between 1RS arms from different sources has been reported [11, 27]. Therefore, to enrich the genetic diversity of 1BL.1RS translocations and to localize genes on 1RS arms by means of meiotic recombination, it is necessary to investigate the consistency of their subtelomeric structure. The *ph1b* mutation was used to induce the homoeologous recombination between wheat 1BS and rye 1RS arms, and an engineered arm 1RS^WW^ was obtained [28, 29]. In this engineered arm, the *Sec-1* locus of 1RS was replaced and the *Gli-B1* and *Glu-B3* loci of 1BS were recovered [28, 29]. The results in this study suggest that the subtelomeric tandem repeats similarity between 1BS and 1RS might be used to improve the efficiency of inducing this kind of engineered arm.

### Unequal recombination of subtelomeric tandem repeats on 1RS

It was reported that tandem repeats pSc119.2, pSc200 and pSc250 present separate domains and formed higher order multimers on rye 1RS arm, and it was proposed that unequal crossing over and homologous recombination contribute to the distribution patterns of these tandem repeats on 1RS arm [9]. During the meiosis of a chimpanzee, partial ectopic paring among several chromosome ends with subtelomeric tandem repeats was observed, and it was postulated that this ectopic association contributed to the ectopic recombination in the regions with satellites and subsequently to the variations of satellite arrays in chimpanzees [30]. These previous studies just inferred that meiotic recombination occurred in the subtelomeric regions enriched with tandem repeats from the evolutionary perspective, but no direct evidence was obtained. In the meiosis of rye, the association of tandem repeat pSc200 between different bivalents in the diplotene stage resulted in the meiotic products with different subtelomeric heterochromatin blocks at the end of meiosis, and this indicated the recombination in the subtelomeric heterochromatin region [31]. However, the state of recombination chromosomes in the next generation was not displayed [31]. In this study, the variation of tandem repeat pSc119.2 and pSc250 on the 1RS arms between two successive generations provides direct evidence that the unequal meiotic recombination can occur in the region with tandem repeats and subsequently induced the variation of these sequences. There were several cellular strategies that prevent meiotic non-allelic homologous recombination (NAHR) from occurring in the regions with tandem repeats, and they include suppression of double strand break (DSB) and promoting the use of an allelic template for the DSB repair [32]. The DSB and meiotic recombination can occur within the maize knob heterochromatin enriched with 180-bp tandem repeat [33]. During meiosis, the normal homolog localization and alignment can inhibit the occurrence of NAHR [34]. So, it is possible that the meiotic recombination occurs in the regions with tandem repeats on 1RS arms. The difference in the composition of the tandem repeats in the subtelomeric regions of 1RS arms might cause the abnormal localization and alignment of homologues during meiosis, leading to unequal meiotic recombination occurred in the regions with pSc119.2 and pSc250 tandem repeats. However, this case only occurred in the progeny of 401Z, suggesting that the different structure existed among the 1RS arms of the three cultivars. In the progenies from the 401L, the 1BL.1RS chromosome with an inversion indicated that the DSB were not repaired in the NOR region and this might be also caused by the different subtelomeric structure of the 1RS arms. During meiosis, the DSB in the rDNA arrays of *Arabidopsis thaliana* were repaired by non-homologous end-joining (NHEJ) rather than by homologous recombination (HR) [35]. Rye 1RS arm is a good model to study whether the DSB repair mechanism of the subtelomeric tandem repeats is the same as that of the NOR.

## Conclusions

Two new olio probes that could display the structural variation of 1RS arms were developed in this study and they were used for investigating the recombination of 1RS arms. The difference in the composition of the tandem repeat pSc200 could affect the recombination of 1RS arm, and led to the unequal recombination of tandem repeats pSc119.2 and pSc250 and an inversion of 1RS arm. These results provide the direct evidence for the meiotic unequal recombination of subtelomeric tandem repeats and a new theoretical basis for the induction of 1RS variation using tandem repeats. In addition, the degree of similarity in the composition of subtelomeric tandem repeats can affect the meiotic recombination of 1RS arms.

## Materials and methods

### Plant materials and cross combinations

Octaploid triticale lines MK were obtained from common wheat (*Triticum aestivum* L.) Mianyang 11 × rye (*Secale cereale* L.) Kustro. Some BC_2_F_4_ seeds were obtained by backcrossing of MK with Mianyang 11 and the wheat-rye 1BL.1RS translocation lines 20T360-2 and 20T401 were identified from the progenies of these BC_2_F_4_ seeds. The wheat-rye 1BL.1RS cultivars Zhou 8425B and Lovrin 10 were kindly provided by State Key Laboratory of Plant Cell and Chromosome Engineering, Institute of Genetics and Developmental Biology, Chinese Academy of Sciences, China. The wheat-rye 1BL.1RS cultivar Chuannong 17 was obtained from the seed store in our laboratory. Six cross combinations were carried out using these 1BL.1RS translocation cultivars/lines as parents (Table 2). Then the seeds of F_2_ generation were obtained by bagged self-fertilization of the F_1_ plants.

**Table 2:** The information about cross combinations

Group 1		Group 2	
Name	Parents	Name	Parents
401Z	20T401 × Zhou 8425B	360Z	20T360-2 × Zhou 8425B
401L	20T401 × Lovrin 10	360L	20T360-2 × Lovrin 10
401C	20T401 × Chuannong 17	360C	20T360-2 × Chuannong 17

### Oligo probes and ND-FISH

Oligo probes Oligo-pSc200 and Oligo-pSc250 were developed according to the methods described by Fu et al. [36]. Two new oligo probes Oligo-119.2-2 and Oligo-TR72 were developed in this study (Table 3). Probe Oligo-119.2-2 was designed according to the tandem repeat family pSc119.2 [12]. A new tandem repeat TR72 from the genomic sequence of rye Lo7 (*Secale cereale* L.) [14] was identified according to the methods described by Tang et al. [37], and the probe Oligo-TR72 was designed using this tandem repeat (Table 3). In addition, oligo probes Oligo-s120.3 [15, 38] and Oligo-1RNOR [39] were also used in this study. Probes Oligo-pSc200 and Oligo-pSc250 produce signals in the telomeric region of the short arm of rye (1RS) [28]. Probe Oligo-TR72 can produce signals in the pericentromeric and intercalary regions of 1RS arm. Probe Oligo-119.2-2 can produce signals in the telomeric and intercalary regions of 1RS arm. Probe Oligo-s120.3 can produce signal in the pericentromeric region of the long arm of wheat chromosome 1B (1BL) [38]. Probe Oligo-1RNOR can be used to specifically identify the nucleolar organizer region (NOR) on 1RS arm [39].

**Table 3:** The information about the newly designed oligo probes

Name of oligo probe	Nucleotide sequence of probe	Base composition of probe	Sequence used for designing probes [Reference]
Oligo-119.2-2	CGAACCCCGGGGTGCG	(GC)_13_(AT)_3_	pSc119.2 [12]
Oligo-TR72	GCTTAGCCGCGAACCCGATTCGCCTAAGTTACAAAAACTACTCCGAGTGAAGAGCAACC	(GC)_30_(AT)_29_	GCTTAGCCGCGAACCCGATTCGCCTAAGTTACAAAAACTACTCCGAGTGAAGAGCAACCCTCTCACTCGGGG [14]

All the oligo probes mentioned above were used for ND-FISH analysis of the root-tip metaphase chromosomes of the materials used in this study. The root-tip metaphase chromosomes were prepared according to the methods described by Han et al. [40]. The oligo probes were 5’-end labeled with Cyanine Dye 5 (Cy5), 6-carboxyfluorescein (6-FAM) or 6-carboxytetramethylrhodamine (TAMRA). The ND-FISH analysis was carried out according to the methods described by Fu et al. [36].

### Calculation of the meiotic recombination frequency of 1RS arm

The recombinant 1RS arms were determined according to the signal patterns of the six probes on 1RS arms. For each cross combination, recombination frequency was calculated as the number of recombinant 1RS arms/total number of 1RS arms × 100%. Then *t*-test was used to determine significant differences in the average recombination rate between group1 and group 2, and this was carried out as described by Girard et al. [41]. All the methods were performed in accordance with the relevant guidelines and regulations.

### Electronic supplementary material

**Additional file 1: Fig. S1.** The first, second and third types of the F_2_ plants from the cross combination group 1. (A-C) The F_1_ type of 1BL.1RS translocations. (A) and (B) are the same cells. (D-I) The parental type of 1BL.1RS translocations. (D) and (E), and (G) and (H) are the same cells, respectively. The probes Oligo-pSc200 (red), Oligo-pSc250 (red), Oligo-119.2-2 (green), Oligo-1RNOR (yellow), Oligo-TR72 (green) and Oligo-s120.3 (green or red) are marked in the figure. Arrows indicate the parental 1BL.1RS chromosomes. Chromosomes were counterstained with DAPI (blue). Scale bar: 10 µm. **Additional file 2: Fig. S2.** The fourth, fifth, sixth and seventh types of the F_2_ plants from the cross combination group 1. (A-C), (D-F), (G-I) and (J-L) represent the fourth, fifth, sixth and seventh type, respectively. (A) and (B), (D) and (E), (G) and (H), and (J) and (K) are the same cells, respectively. The probes Oligo-pSc200 (red), Oligo-pSc250 (red), Oligo-119.2-2 (green), Oligo-1RNOR (yellow), Oligo-TR72 (green) and Oligo-s120.3 (green or red) are marked in the figure. Arrows indicate the parental or recombinant 1BL.1RS chromosomes. Chromosomes were counterstained with DAPI (blue). Scale bar: 10 µm. **Additional file 3: Fig. S3.** The eighth, ninth and tenth types of the F_2_ plants from the cross combination group 1. (A-C), (D-F) and (G-I) represent the eighth, ninth and tenth type, respectively. (A) and (B), (D) and (E), and (G) and (H) are the same cells, respectively. The probes Oligo-pSc200 (red), Oligo-pSc250 (red), Oligo-119.2-2 (green), Oligo-1RNOR (yellow), Oligo-TR72 (green) and Oligo-s120.3 (green or red) are marked in the figure. Arrows indicate the parental or recombinant 1BL.1RS chromosomes. Chromosomes were counterstained with DAPI (blue). Scale bar: 10 µm. **Additional file 4: Fig. S4.** The eleventh, twelfth and thirteenth types of the F_2_ plants from the cross combination group 1. (A-C), (D-F) and (G-I) represent the eleventh, twelfth and thirteenth type, respectively. (A) and (B), (D) and (E), and (G) and (H) are the same cells, respectively. The probes Oligo-pSc200 (red), Oligo-pSc250 (red), Oligo-119.2-2 (green), Oligo-1RNOR (yellow), Oligo-TR72 (green) and Oligo-s120.3 (green or red) are marked in the figure. Arrows indicate the parental or recombinant 1BL.1RS chromosomes. Chromosomes were counterstained with DAPI (blue). Scale bar: 10 µm. **Additional file 5: Fig. S5.** The first, second and third types of the F_2_ plants from the cross combination group 2. (A-C) The F_1_ type, (A) and (B) are the same cells. (D-I) The parental type, (D) and (E), and (G) and (H) are the same cells, respectively. The probes Oligo-pSc200 (red), Oligo-pSc250 (red), Oligo-119.2-2 (green), Oligo-1RNOR (yellow), Oligo-TR72 (green) and Oligo-s120.3 (red or yellow) are marked in the figure. Arrows indicate the parental 1BL.1RS chromosomes. Chromosomes were counterstained with DAPI (blue). Scale bar: 10 µm. **Additional file 6: Fig. S6.** The fourth, fifth, sixth and seventh types of the F_2_ plants from the cross combination group 2. (A-C), (D-F), (G-I) and (J-L) represent the fourth, fifth, sixth and seventh type, respectively. (A) and (B), (D) and (E), (G) and (H), and (J) and (K) are the same cells, respectively. The probes Oligo-pSc200 (red), Oligo-pSc250 (red), Oligo-119.2-2 (green), Oligo-1RNOR (yellow), Oligo-TR72 (green) and Oligo-s120.3 (green or red) are marked in the figure. Arrows indicate the parental or recombinant 1BL.1RS chromosomes. Chromosomes were counterstained with DAPI (blue). Scale bar: 10 µm. **Additional file 7: Fig. S7.** The eighth, ninth and tenth types of the F_2_ plants from the cross combination group 2. (A-C), (D-F) and (G-I) represent the eighth, ninth and tenth type, respectively. (A) and (B), (D) and (E), and (G) and (H) are the same cells, respectively. The probes Oligo-pSc200 (red), Oligo-pSc250 (red), Oligo-119.2-2 (green), Oligo-1RNOR (yellow), Oligo-TR72 (green) and Oligo-s120.3 (green or red) are marked in the figure. Arrows indicate the parental or recombinant 1BL.1RS chromosomes. Chromosomes were counterstained with DAPI (blue). Scale bar: 10 µm.

## Data Availability

The datasets are available in this manuscript and the materials used and/or analyzed during the current study are available from the corresponding author on reasonable request.

## Abbreviations

Oligonucleotide: oligo
Non-denaturing fluorescence *in situ* hybridization: ND-FISH
