# Supplementary material for: The different subtelomeric structure among 1RS arms in wheat-rye 1BL.1RS translocations affecting their meiotic recombination and inducing their structural variation
Source: BMC Genomics. 2023 Aug 11;24:455. doi: 10.1186/s12864-023-09525-9 (PMC10416389; doi:10.1186/s12864-023-09525-9)
Supplement: Supplementary file 1 — Additional file 1: Fig. S1. The first, second and third types of the F2 plants from the cross combination group 1. (A-C) The F1 type of 1BL.1RS translocations. (A) and (B) are the same cells. (D-I) The parental type of 1BL.1RS translocations. (D) and (E), and (G) and (H) are the same cells, respectively. The probes Oligo-pSc200 (red), Oligo-pSc250 (red), Oligo-119.2-2 (green), Oligo-1RNOR (yellow), Oligo-TR72 (green) and Oligo-s120.3 (green or red) are marked in the figure. Arrows indicate the parental 1BL.1RS chromosomes. Chromosomes were counterstained with DAPI (blue). Scale bar: 10 µm. Additional file 2: Fig. S2. The fourth, fifth, sixth and seventh types of the F2 plants from the cross combination group 1. (A-C), (D-F), (G-I) and (J-L) represent the fourth, fifth, sixth and seventh type, respectively. (A) and (B), (D) and (E), (G) and (H), and (J) and (K) are the same cells, respectively. The probes Oligo-pSc200 (red), Oligo-pSc250 (red), Oligo-119.2-2 (green), Oligo-1RNOR (yellow), Oligo-TR72 (green) and Oligo-s120.3 (green or red) are marked in the figure. Arrows indicate the parental or recombinant 1BL.1RS chromosomes. Chromosomes were counterstained with DAPI (blue). Scale bar: 10 µm. Additional file 3: Fig. S3. The eighth, ninth and tenth types of the F2 plants from the cross combination group 1. (A-C), (D-F) and (G-I) represent the eighth, ninth and tenth type, respectively. (A) and (B), (D) and (E), and (G) and (H) are the same cells, respectively. The probes Oligo-pSc200 (red), Oligo-pSc250 (red), Oligo-119.2-2 (green), Oligo-1RNOR (yellow), Oligo-TR72 (green) and Oligo-s120.3 (green or red) are marked in the figure. Arrows indicate the parental or recombinant 1BL.1RS chromosomes. Chromosomes were counterstained with DAPI (blue). Scale bar: 10 µm. Additional file 4: Fig. S4. The eleventh, twelfth and thirteenth types of the F2 plants from the cross combination group 1. (A-C), (D-F) and (G-I) represent the eleventh, twelfth and thirteenth type, respectiv [file 12864_2023_9525_MOESM1_ESM.docx]

Supplementary Figure legends

Additional files

**Additional file 1: Fig. S1.** The first, second and third types of the F_2_ plants from the cross combination group 1. (A-C) The F_1_ typeof 1BL.1RS translocations . (A) and (B) are the same cells. (D-I) The parental type of 1BL.1RS translocations. (D) and (E), and (G) and (H) are the same cells, respectively. The probes Oligo-pSc200 (red), Oligo-pSc250 (red), Oligo-119.2-2 (green), Oligo-1RNOR (yellow), Oligo-TR72 (green) and Oligo-s120.3 (green or red) are marked in the figure. Arrows indicate the parental 1BL.1RS chromosomes. Chromosomes were counterstained with DAPI (blue). Scale bar: 10 μm.

**Additional file 2: Fig. S2.** The fourth, fifth, sixth and seventh types of the F_2_ plants from the cross combination group 1. (A-C), (D-F), (G-I) and (J-L) represent the fourth, fifth, sixth and seventh type, respectively. (A) and (B), (D) and (E), (G) and (H), and (J) and (K) are the same cells, respectively. The probes Oligo-pSc200 (red), Oligo-pSc250 (red), Oligo-119.2-2 (green), Oligo-1RNOR (yellow), Oligo-TR72 (green) and Oligo-s120.3 (green or red) are marked in the figure. Arrows indicate the parental or recombinant 1BL.1RS chromosomes. Chromosomes were counterstained with DAPI (blue). Scale bar: 10 μm.

**Additional file 3: Fig. S3.** The eighth, ninth and tenth types of the F_2_ plants from the cross combination group 1. (A-C), (D-F) and (G-I) represent the eighth, ninth and tenth type, respectively. (A) and (B), (D) and (E), and (G) and (H) are the same cells, respectively. The probes Oligo-pSc200 (red), Oligo-pSc250 (red), Oligo-119.2-2 (green), Oligo-1RNOR (yellow), Oligo-TR72 (green) and Oligo-s120.3 (green or red) are marked in the figure. Arrows indicate the parental or recombinant 1BL.1RS chromosomes. Chromosomes were counterstained with DAPI (blue). Scale bar: 10 μm.

**Additional file 4: Fig. S4.** The eleventh, twelfth and thirteenth types of the F_2_ plants from the cross combination group 1. (A-C), (D-F) and (G-I) represent the eleventh, twelfth and thirteenth type, respectively. (A) and (B), (D) and (E), and (G) and (H) are the same cells, respectively. The probes Oligo-pSc200 (red), Oligo-pSc250 (red), Oligo-119.2-2 (green), Oligo-1RNOR (yellow), Oligo-TR72 (green) and Oligo-s120.3 (green or red) are marked in the figure. Arrows indicate the parental or recombinant 1BL.1RS chromosomes. Chromosomes were counterstained with DAPI (blue). Scale bar: 10 μm.

**Additional file 5: Fig. S5.** The first, second and third types of the F_2_ plants from the cross combination group 2. (A-C) The F_1_ type, (A) and (B) are the same cells. (D-I) The parental type, (D) and (E), and (G) and (H) are the same cells, respectively. The probes Oligo-pSc200 (red), Oligo-pSc250 (red), Oligo-119.2-2 (green), Oligo-1RNOR (yellow), Oligo-TR72 (green) and Oligo-s120.3 (red or yellow) are marked in the figure. Arrows indicate the parental 1BL.1RS chromosomes. Chromosomes were counterstained with DAPI (blue). Scale bar: 10 μm.

**Additional file 6: Fig. S6.** The fourth, fifth, sixth and seventh types of the F_2_ plants from the cross combination group 2. (A-C), (D-F), (G-I) and (J-L) represent the fourth, fifth, sixth and seventh type, respectively. (A) and (B), (D) and (E), (G) and (H), and (J) and (K) are the same cells, respectively. The probes Oligo-pSc200 (red), Oligo-pSc250 (red), Oligo-119.2-2 (green), Oligo-1RNOR (yellow), Oligo-TR72 (green) and Oligo-s120.3 (green or red) are marked in the figure. Arrows indicate the parental or recombinant 1BL.1RS chromosomes. Chromosomes were counterstained with DAPI (blue). Scale bar: 10 μm.

**Additional file 7: Fig. S7.** The eighth, ninth and tenth types of the F_2_ plants from the cross combination group 2. (A-C), (D-F) and (G-I) represent the eighth, ninth and tenth type, respectively. (A) and (B), (D) and (E), and (G) and (H) are the same cells, respectively. The probes Oligo-pSc200 (red), Oligo-pSc250 (red), Oligo-119.2-2 (green), Oligo-1RNOR (yellow), Oligo-TR72 (green) and Oligo-s120.3 (green or red) are marked in the figure. Arrows indicate the parental or recombinant 1BL.1RS chromosomes. Chromosomes were counterstained with DAPI (blue). Scale bar: 10 μm.
